# Variation of the Three-Dimensional Femoral J-Curve in the Native Knee

**DOI:** 10.3390/jpm11070592

**Published:** 2021-06-23

**Authors:** Sonja A. G. A. Grothues, Klaus Radermacher

**Affiliations:** Chair of Medical Engineering, Helmholtz Institute for Biomedical Engineering, RWTH Aachen University, 52074 Aachen, Germany; radermacher@hia.rwth-aachen.de

**Keywords:** native knee morphology, femoral J-Curve, principal component analysis, geometric parameter analysis

## Abstract

The native femoral J-Curve is known to be a relevant determinant of knee biomechanics. Similarly, after total knee arthroplasty, the J-Curve of the femoral implant component is reported to have a high impact on knee kinematics. The shape of the native femoral J-Curve has previously been analyzed in 2D, however, the knee motion is not planar. In this study, we investigated the J-Curve in 3D by principal component analysis (PCA) and the resulting mean shapes and modes by geometric parameter analysis. Surface models of 90 cadaveric femora were available, 56 male, 32 female and two without respective information. After the translation to a bone-specific coordinate system, relevant contours of the femoral condyles were derived using virtual rotating cutting planes. For each derived contour, an extremum search was performed. The extremum points were used to define the 3D J-Curve of each condyle. Afterwards a PCA and a geometric parameter analysis were performed on the medial and lateral 3D J-Curves. The normalized measures of the mean shapes and the aspects of shape variation of the male and female 3D J-Curves were found to be similar. When considering both female and male J-Curves in a combined analysis, the first mode of the PCA primarily consisted of changes in size, highlighting size differences between female and male femora. Apart from changes in size, variation regarding aspect ratio, arc lengths, orientation, circularity, as well as regarding relative location of the 3D J-Curves was found. The results of this study are in agreement with those of previous 2D analyses on shape and shape variation of the femoral J-Curves. The presented 3D analysis highlights new aspects of shape variability, e.g., regarding curvature and relative location in the transversal plane. Finally, the analysis presented may support the design of (patient-specific) femoral implant components for TKA.

## 1. Introduction

The sagittal shape of the femoral condyles, which is often referred to as J-Curve, is known to be a significant determinant of knee biomechanics [1]. Similarly, in total knee arthroplasty (TKA), the J-Curve of the femoral component is reported to have a high impact on knee kinematics [2] and its relevance is reflected in various implant design philosophies, including single-, dual-, and multi-radius designs. The medial and lateral J-Curve approximate the contours being in contact with the tibial plateaus and thereby they are highly relevant for tibiofemoral articulation. Therefore, the J-Curve is related to relevant motion phenomena of the native knee, such as femoral rollback and medial pivot [1,3]. Those are linked to flexion range of motion [4] and patient satisfaction in general [5]. In addition, the J-Curve or rather its alteration is highly relevant for ligament strain and tension as well as for the resulting tibiofemoral contact forces. With a ligament stiffness of 60–80 N/mm of medial and lateral collateral ligaments (MCL/LCL) [6,7], a local condylar offset compared to the native J-Curve of only 1 mm will result either in 60–80 N additional lateral and medial tibiofemoral contact force and increased ligament strain; or in ligament relaxation and potential (mid-flexion) instability. In addition, first structural damage is occurring in ligaments from about 5% strain [8]. With an assumed average length of the MCL(LCL) of 100(60) mm, a medial (lateral) offset limit would be 5(3) mm (corresponding to 5% maximum strain) which would result in additional medial (lateral) forces of ~300–400(180–240)N for an average knee. Taking into account, that knee arthroplasty should not extend ligament strain up to the limits of structural damage, and that loads of 10 N (corresponding to less than 1 mm offset) already activate afferent nerves from receptors in the ligaments triggering the knee joint stabilizing muscles (Sojka et al., 1991), we assume, that local J-Curve offset limits would have to be reduced to the range of 1–2 mm maximum. This is in agreement with literature regarding recommendations for varus-valgus laxity between 0.5 and 1 mm for extension and 0.7–1.2 mm for flexion [9].

Consequently, the analysis of the native femoral J-Curve is essential for a better understanding of native knee biomechanics and for optimizing the femoral implant component design in TKA. Previous analyses of the femoral J-Curve have focused on its 2D shape in one specific cutting plane or through projection. Most studies used geometrical primitives such as ellipses and circles and fitted them to the respective 2D J-Curve contours for investigation [10,11,12,13,14,15]. In a previous study, we evaluated the variation in the native femoral J-Curve by principal component analysis (PCA), enabling a more comprehensive investigation of the shape variation [16]. However, due to the 3D nature of knee motion, the restriction to a 2D evaluation remained a limitation of this study. Hiss and Schwerbrock [17] analyzed the condylar extremum points of a cadaveric knees in 3D, by a comprehensive manual analysis. A limitation of their labor-intensive method is that it is not applicable to large sample sizes. A limitation of their analysis was that they neglected the J-Curve’s orientation with regard to the mechanical axis, whereby a relevant amount of variation was neglected. Other authors analyzed the tibiofemoral process of contacts e.g., by finite element simulations [18], but did not evaluate the derived points regarding shape variation.

The aim of this study was to investigate the 3D femoral J-Curve of the native knee by principal component and geometric parameter analysis.

## 2. Materials and Methods

### 2.1. Patient Datasets

Bone surface models of 90 cadaveric femora, which have been segmented semiautomatically (control by experts) from CT data (voxel size: 0.49/0.53 mm), were provided by ConforMIS (ConforMIS Inc., Billerica, MA, USA). Of the 90 cadavers, 56 were male, 32 female, and for two no gender information was available. The bone models showed no osteophytes or other signs of osteoarthritis. All further processing was performed in semiautomatic self-written MATLAB scripts (Version R2018b, The MathWorks, Inc., Natick, MA, USA).

### 2.2. Contour Derivation

**Figure 1:**
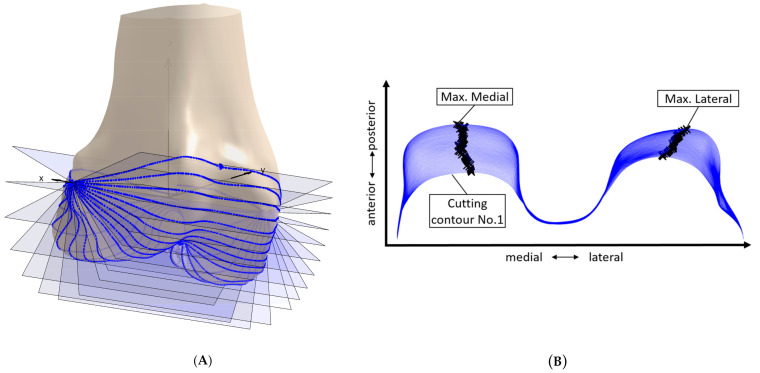
Elements of the process of contour derivation. (**A**) Example femur with rotating cutting planes for the derivation of cutting contour (note: only 18 cutting planes displayed here, to enable better visualization of the individual planes). (**B**) Cutting contours (blue) and extrema (black) for cutting planes 1 to 63.

First, the bone models were transferred to a bone-specific coordinate system [19]. Left femora were mirrored. In order to determine relevant bony contours, the concept of rotating cutting planes was used, which has been previously applied in the context of surface parametrization [19,20]. The concept is depicted in Figure 1A. The transepicondylar axis was used as origin of the cutting planes. Overall 300 cutting planes between extremum points of the articulating areas on the condyles and the trochlea were used (note Figure 1A shows only 18 cutting planes for better visibility of the individual cutting planes). For each cutting plane a cutting contour was derived. Subsequently, for each contour an extremum search was performed, as it can be seen in Figure 1B. Therefore, the contours were transformed to the x-y plane, and extrema (maxima) regarding the y-axis were identified. For the contours defined by the extrema, a curvature analysis was performed, in order to determine the boundaries of the articulating area, according to Li et al. [13]. The contours were then cut accordingly and interpolated by 300 equidistant points.

### 2.3. Principal Component Analysis

Principal component analysis (PCA) is a mathematical method, which is used for reducing dimensionality of multivariate datasets. In PCA, the principal components are calculated, which represent the directions along which the data varies the most. The principal components can be derived by calculating the eigenvectors of the covariance matrix, and they are ordered according to the amount of variance they account for [21].

In the present study, PCA was used to identify dominant patterns of contour variation. PCA requires corresponding data points (landmarks) between the subjects. This is enabled by the use of a consistent bone-specific coordinate system for the contour derivation, and the standardized definition of boundary points. The PCA was performed combined on both the medial and lateral femoral 3D J-Curves. The analysis was performed according to Shlens [22]. The principal modes were defined according to Stegmann and Gomez [23]. The female and male cadavers were analyzed separately as well as combined, in order to evaluate differences in gender.

### 2.4. Geometric Parameter Analysis

**Figure 2:**
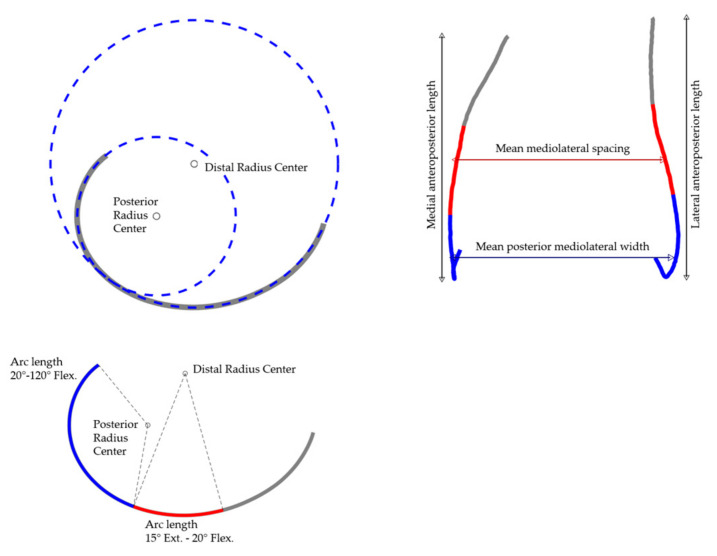
Visualization of the geometric parameter analysis on the example of the mean shape (combined population).

A geometric parameter analysis was applied to the mean shape as well as to the first five modes. General size parameters, arc lengths, radii describing the curvature, and the mean and maximum local condylar offsets were considered. The parameters are listed and described in detail in Table 1. In addition, the parameters are displayed in Figure 2. Changes in parameter measures originating from the modes were quantified in absolute deviations and in percent.

## 3. Results

**Figure 3:**
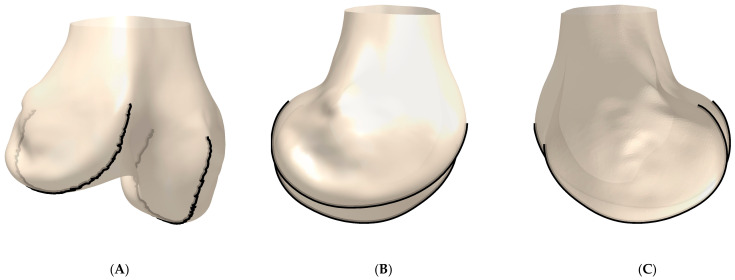
Example of the derived 3D J-Curve contours. (**A**) Anterior/lateral-posterior/medial view. (**B**) Lateral-medial view. (**C**) Medial-lateral view.

**Figure 4:**
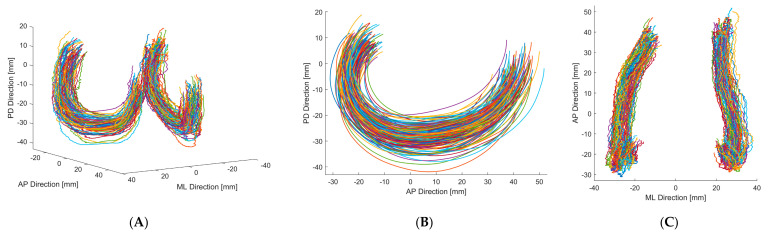
3D J-Curve contours of both genders. (**A**) Anterior/lateral-posterior/medial view. (**B**) Lateral-medial view. (**C**) Superior-inferior view.

In total, 85 of the 90 cadaver cases could be processed without errors (54 male, 29 females, 2 without gender information). Figure 3 shows an example of the derived contours of one femur, together with the respective bone model. An overview of all derived 3D J-Curves is given in Figure 4.

The mean shapes of the male, female and combined population differed regarding the morphological measures considered (Table 2). However, after normalization of the measures according to their direction of measurement (mediolateral measures by the posterior mediolateral width, anteroposterior measures by the anteroposterior size) as suggested by Asseln et al. [27], those normalized measures were comparable for the male, female and combined population, as it can be seen in Table 2.

**Figure 5:**
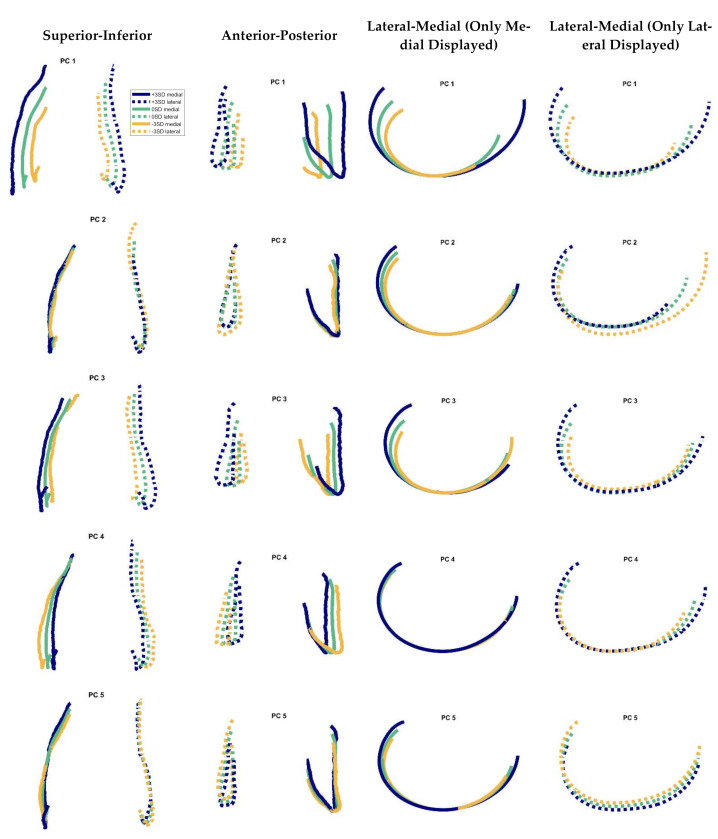
Modes 1–5 of the cadavers’ 3D J-Curves in different views. Solid line: medial, dashed line: lateral. 3SD = 3 standard deviations. All contours were oriented to their most distal point in proximodistal direction, for better comparison of the respective variance. Variation explained by the modes 1–5: 31.5, 23.4, 20.1, 7.4, and 5.5%, respectively.

The results of the separate PCA of female and male 3D J-Curves showed similarities regarding the aspects of shape variations (e.g., arc lengths, orientation, aspect ratio). For the combined analysis (Figure 5), the first mode consisted almost solely of changes in size, highlighting size differences between female and male femora. Apart from this first mode, the aspects of shape variation were similar for all analyses. Due to similarities in normalized measures of the mean shapes and in the aspects of shape variation, in the following only the detailed results of the combined analysis of both genders are presented.

Figure 5 shows the PCA results regarding the first five modes. The percentage of variation explained by modes 1–5 were 31.5, 23.4, 20.1, 7.4, and 5.5%, respectively (sum: 87.8%). In Table 3 the results of the respective geometric parameter analysis are presented. The first mode involved changes in size, which lead to an increase of all parameters in the geometric parameter analysis, when adding 3 standard deviations to the mean shape (Table 3). Furthermore, for the medial side, also slight changes in 3D J-Curve orientation were associated. With the second mode, the most prominent changes were seen regarding the anterior region of the lateral J-Curve. For the medial side, only slight changes in curvature and size were observed. The third mode consisted of changes in medial J-Curve orientation, in lateral J-Curve size and in mediolateral width. The fourth mode primarily represented changes in aspect ratio. The fifth mode mostly consisted of changes in relative location of the medial vs. the lateral 3D J-Curve.

## 4. Discussion

In contrast to previous analyses on the 2D J-Curve shape, the analysis presented enabled the consideration of shape and shape variation in the transversal plane. Compared to a previous study by Hiss and Schwerbrock [17] on femoral J-Curves in 3D, the presented analysis was performed semiautomatically, which enabled the processing of a higher number of femora.

Similar aspects of shape variation of the femoral 3D J-Curves were found in men and in women. The amount of variation explained by changes in size was higher for the combined than for the gender-specific analyses. This is reasonable, as men in general have larger knees compared to women [27]. Hence, the combination of both genders probably is the reason for the increased variability in size.

For the combined analyses, the identified radii of the 3D J-Curve’s mean shape are comparable to those of previous studies on the 2D J-Curve [11,12,15,27]. Most of the parameter values derived in this study are also comparable to a previous study on the 2D J-Curve by our group [16]. However, a relevant difference regarding the AP length of the medial J-Curve can be seen. The medial 3D J-Curve shows a higher AP length compared to the medial 2D J-Curve. This may be explained by the distribution of the medial condyle’s extremum points in the transversal plane (Figure 4C). The extrema of both condyles do not lie in a single sagittal plane. Especially the medial extrema rather form a curve. As for the 2D J-Curve derivation a single sagittal cutting plane was used, parts of the medial J-Curve may have been neglected. This effect may also be present to a lower extent for the lateral side, as the lateral 2D J-Curve is also slightly smaller in AP direction compared to the lateral 3D J-Curve.

The general relevance of morphological parameters for knee kinematics has been shown in a previous study by our group [28]. In the first degrees of flexion, the share of rolling vs. gliding of the femur on the tibia is estimated to be 1:2 [29]. Afterwards, the motion can be characterized as primarily gliding (in late flexion: rolling/gliding 1:4) [29]. Therefore, the arc length in the beginning of flexion is of higher functional relevance, as it represents the primary running surface of the respective condyle and thereby influences the range of tibiofemoral anterior-posterior translation and internal-external rotation. In the PCA results, changes in the distal arc length differed between the medial and lateral side and were even counteracting for modes 4 and 5 (Table 3).

In our study, mean absolute condylar offsets in the range of 2.09–9.36 mm and local maximum offsets in the range of 2.61–16.0 mm were found. Those exceed the derived offset limits of 1–2 mm. It has to be noted that with ±3 standard deviations, a wide range of variation was considered. However, every patient needs to be provided with an adequate implant. In addition, all mean offsets were larger than 2 mm, suggesting that a relevant share of the patient population may receive an implant with local condylar offsets exceeding those limits. Some of the variation regarding size and aspect ratio is accounted for by different implant sizes and narrow/standard implant versions. Remaining variation, however, is not accounted for with standard implants.

### Limitations

The study presented involved limitations. First, the start and end points of the J-Curves were determined automatically by curvature analysis and not by a visual inspection of the clinical images. However, this automation was necessary in order to enable the processing of a large number of cases.

Second, the use of an extremum search still is an approximation of an actual course of tibiofemoral contact points on the femur. However, the extremum search used in this study identified relevant points on the contours, which correspond to contact points of femoral and tibial implant components in TKA. Therefore, we believe the contours to be of relevance for implant design.

Third, the database is limited to 90 cases of unknown ethnicity. Further analyses are necessary to investigate more cases and evaluate differences between ethnicities. In addition, this study is restricted to the analysis of the femur. Future analyses should also investigate the tibial sagittal contours and the patellofemoral contact (native vs. alloplastic).

Lastly, this study only addresses implant design as one factor with influence on clinical outcome and patient satisfaction in TKA. There are many other potentially relevant influencing factors, such as surgical technique, muscular and ligamentous situation, patient’s expectations, etc. However, by optimizing the J-Curve “fit”, the potential for superior outcomes may be enabled.

## 5. Conclusions

The results of this study suggest that variation in the native femoral 3D J-Curves does not only involve scaling and aspect ratio changes, but other aspects such as changes in curvature or circularity, arc lengths, and relative location. Current OTS implant manufacturers offer various implant sizes (i.e., scaling only) as well as narrow and wide implants, accounting for differences in size and in aspect ratio. Differences in other aspects such as in curvature are not accounted for so far. The industry aims at a better restoration of knee morphology, e.g., by introducing more sizes or gender-specific implants. Hence, for future implant systems it might be valuable not only to consider narrow and standard versions but, e.g., high and low curvature implants as well as versions with different offsets. Taking into account the importance of shape mismatches along the articulating surfaces [9,30] as well as the discrepancy between actual implant designs and patient specific J-Curves [16], the number of additional sizes needed potentially will be very high. Against this background, we agree to the conclusion of Delport et al., that another way could be to customize the implant design to each patient individually [9]. In such cases, however, additional attention to force distribution and contact areas between implant surfaces may be needed, depending upon factors such as the nature and degree of the customization of the implant design. 

Due to the relevance of bone morphology for active kinematics, related soft tissue strains and for the overall clinical outcome [1,3,4,5], patient specific 3D J Curves derived from individual image data could be used to evaluate therapeutic options (OTS implants vs. patient specific implants (intrinsically reflecting patient specific J-Curve shape)) and to decide for an adequate match for each patient individually.

## Figures and Tables

**Table 1:** Description of the parameters considered in the geometric parameter analysis. Parameters are either defined for the combined overall shape of both J-Curves or individually for the medial and lateral side (column: overall/medial and lateral).

Parameter Name	Overall/Medial and Lateral	Unit	Description
Mean distal ML spacing	Overall	mm	Mean mediolateral distance of the distal points of the lateral/medial 3D J-Curve (15° of extension to 20° of flexion, reference: radius of the circle fitted to the distal portion of the condyles). Inspired by Walker [24].
Mean posterior ML width	Overall	mm	Mean mediolateral distance of the posterior points of the lateral/medial 3D J-Curve (20°–120° of flexion, reference: radius of the circle fitted to the posterior portion of the condyles). Inspired by Mahfouz [25].
AP length	Medial and lateral	mm	Anteroposterior length of the medial/lateral 3D J-Curve.
Distal radius	Medial and lateral	mm	Radius of the circle fitted to the distal portion of the medial/lateral 3D J-Curve. The calculation was performed according to Nuno and Ahmed [15] and is described in more detail in Asseln et al. [26].
Posterior radius	Medial and lateral	mm	Radius of the circle fitted to the posterior portion of the medial/lateral 3D J-Curve. The calculation was performed according to Nuno and Ahmed [15] and is described in more detail in Asseln et al. [26].
Functional arc length	Medial and lateral	mm	Arc length of the medial/lateral 3D J-Curve between 15° of extension until 120° of flexion (reference: center of the circle fitted to the distal/posterior portion of the condyles).
Arc length 15° Ext.–20° Flex.	Medial and lateral	mm	Arc length of the medial/lateral 3D J-Curve between 15° of extension until 20° of flexion (reference: center of the circle fitted to the distal portion of the condyles).
Arc length 20°–120° Flex.	Medial and lateral	mm	Arc length of the medial/lateral 3D J-Curve between 20° until 120° of flexion (reference: center of the circle fitted to the distal/ posterior portion of the condyles).
Mean abs. deviation	Medial and lateral	mm	Mean absolute deviation (mean condylar offset) regarding anteroposterior and proximodistal direction.
Max abs. deviation	Medial and lateral	mm	Maximum absolute deviation (maximum condylar offset) regarding anteroposterior and proximodistal direction.

**Table 2:** Results of the geometric parameter analysis: measures of the mean shapes of the male, female, and combined population are listed. In addition, normalized measures are given in brackets.

Parameter (Normalized by ML/AP)	Mean ML Spacing	Mean Posterior ML Width		AP Length	Distal Radius	Posterior Radius	Funct. Arc Length	Arc Length 15°Ext.–20° Flex.	Arc Length 20°–120° Flex.
**Mean shape (combined)**	51.2 mm (0.95)	53.7 mm	Lateral	64.2 mm (0.99)	48.8 mm (0.75)	20.3 mm (0.31)	67.4 mm (1.04)	32.5 mm (0.50)	34.9 mm (0.54)
			Medial	60.1 mm (0.93)	35.1 mm (0.54)	19.3 mm (0.30)	67.5 mm (1.04)	22.8 mm (0.35)	44.7 mm (0.69)
**Mean shape (Male)**	53.7 mm (0.96)	56.1 mm	Lateral	66.9 mm (0.99)	50.5 mm (0.75)	21.4 mm (0.32)	69.7 mm (1.03)	33.7 mm (0.50)	35.9 mm (0.53)
			Medial	62.8 mm (0.93)	36.9 mm (0.55)	20.2 mm (0.30)	70.1 mm (1.04)	23.9 mm (0.36)	46.2 mm (0.69)
**Mean shape (Female)**	46.2 mm (0.94)	49.1 mm	Lateral	60.5 mm (0.99)	46.9 mm (0.77)	18.6 mm (0.31)	64.0 mm (1.05)	30.9 mm (0.51)	33.2 mm (0.54)
			Medial	55.2 mm (0.91)	31.5 mm (0.52)	17.7 mm (0.29)	62.9 mm (1.03)	20.4 mm (0.33)	42.5 mm (0.70)

**Table 3:** Results of the geometric parameter analysis: Effect sizes for the first five modes are listed (+3SD). Deviations with regard to the mean shape are quantified in millimeter and in percent. Changes exceeding predefined limits are highlighted (color code below). Abbreviations: AP = anteroposterior, ML = mediolateral.

Parameter		Mean ML Spacing	Mean Posterior ML Width		AP Length	Distal Radius	Posterior Radius	Funct. Arc Length	Arc Length 15°Ext.–20° Flex.	Arc Length 20°–120° Flex.	Mean Abs. Deviation
**Mode**	**1**	11.79 mm (23.0%)	10.96 mm (20.4%)	Lateral	12.15 mm (18.9%)	7.31 mm (15%)	4.85 mm (23.9%)	12.3 mm (18.3%)	5.42 mm (16.7%)	6.88 mm (19.7%)	5.34 mm
				Medial	16.84 mm (28%)	8.31 mm (23.7%)	5.26 mm (27.2%)	13.35 mm (19.8%)	6.01 mm (26.4%)	7.35 mm (16.4%)	8.81 mm
	**2**	3.71 mm (7.2%)	−0.3 mm (−0.6%)	Lateral	−7.11 mm (−11.1%)	2.33 mm (4.8%)	0.54 mm (2.7%)	10.85 mm (16.1%)	1.56 mm (4.8%)	9.29 mm (26.6%)	9.36 mm
				Medial	3.4 mm (5.7%)	1.99 mm (5.7%)	0.87 mm (4.5%)	6.84 mm (10.1%)	1.72 mm (7.6%)	5.12 mm (11.4%)	4.56 mm
	**3**	4.72 mm (9.2%)	7.7 mm (14.3%)	Lateral	6.59 mm (10.3%)	9 mm (18.4%)	3.44 mm (16.9%)	16.2 mm (24%)	7.76 mm (23.9%)	8.44 mm (24.2%)	4.37 mm
				Medial	3.32 mm (5.5%)	4.08 mm (11.6%)	2.53 mm (13.1%)	11.75 mm (17.4%)	2.82 mm (12.4%)	8.93 mm (20%)	7.08 mm
	**4**	−4.72 mm (−9.2%)	−6.31 mm (−11.7%)	Lateral	6.49 mm (10.1%)	7.97 mm (16.3%)	1.75 mm (8.6%)	12.93 mm (19.2%)	6.17 mm (19%)	6.76 mm (19.4%)	3.86 mm
				Medial	2.63 mm (4.4%)	−0.24 mm (−0.7%)	0.29 mm (1.5%)	3.45 mm (5.1%)	−0.07 mm (−0.3%)	3.52 mm (7.9%)	2.09 mm
	**5**	−0.56 mm (−1.1%)	−0.72 mm (−1.3%)	Lateral	−0.49 mm (−0.8%)	−3.33 mm (−6.8%)	−0.17 mm (−0.8%)	−4.02 mm (−6%)	−1.61 mm (−4.9%)	−2.4 mm (−6.9%)	2.97 mm
				Medial	4.48 mm (7.4%)	3.28 mm (9.3%)	0.76 mm (3.9%)	6.22 mm (9.2%)	2.39 mm (10.5%)	3.83 mm (8.6%)	3.66 mm

**Color code: Deviations:**≥ ±10%: ■**|**≥ ±20%: ■**Mean abs. deviation:**≥2 mm: ■ | ≥5 mm: ■.
